# A minimal deterministic model reveals integration of spindle assembly and position checkpoints in mitosis

**DOI:** 10.1038/s41598-025-11673-9

**Published:** 2025-07-22

**Authors:** Bashar Ibrahim

**Affiliations:** 1 Department of Mathematics & Natural Sciences and Centre for Applied Mathematics & Bioinformatics, Gulf University for Science and Technology, 32093 Hawally, Kuwait; 2 Department of Mathematics and Computer Science, Friedrich Schiller University Jena, Fürstengraben, 07743 Jena, Germany; 3 European Virus Bioinformatics Center, Leutragraben 1, 07743 Jena, Germany

**Keywords:** Ultrasensitive Coordination of SAC–SPOC Checkpoints, Computational biology and bioinformatics, Systems biology

## Abstract

The spindle assembly checkpoint (SAC) and spindle position checkpoint (SPOC) are essential surveillance systems that ensure accurate chromosome segregation and proper spindle orientation during mitosis. While their individual mechanisms have been extensively studied, their functional integration remains poorly understood. Here, I present a minimal deterministic mathematical model that captures key interactions between SAC and SPOC, incorporating central components such as Mad2, Cdc20, APC/C, Bfa1, Bub2, Tem1, Kin4, and the mitotic kinase Cdc5. The analysis identifies four distinct operational regimes—checkpoint silence, SAC-dominant arrest, SPOC-dominant arrest, and dual-checkpoint arrest—providing a conceptual framework for how cells respond to various spindle defects. This work represents the first comprehensive mathematical framework that integrates these two critical checkpoint systems. The model includes tension-sensitive feedback and demonstrates that deterministic dynamics alone can generate ultrasensitive, switch-like checkpoint responses—without requiring stochastic fluctuations or spatial complexity. Simulations reproduce key experimental observations, including the effects of *in vitro* mutations in core components and the rheostat-like degradation dynamics of Securin and Cyclin B. Notably, the model exhibits dual regulatory behavior: a bistable toggle switch within the SAC core driven by autocatalytic feedback, and a graded, rheostat-like output at the level of checkpoint satisfaction. This reconciles seemingly contradictory observations of discrete molecular switches with continuous cellular responses. Together, these findings offer a simplified yet predictive framework for dissecting mitotic checkpoint integration and lay the groundwork for future experimental and theoretical studies of SAC–SPOC coordination.

## Introduction

Accurate chromosome segregation and proper spindle orientation during mitosis are essential for maintaining genomic stability and ensuring faithful cell division. Two key regulatory mechanisms safeguard these processes: the spindle assembly checkpoint (SAC) and the spindle position checkpoint (SPOC), which monitor kinetochore attachment and spindle alignment, respectively^1,2^.

The SAC is a highly conserved surveillance system across eukaryotes, from yeast to humans, underscoring its critical role in controlling anaphase onset^3^. In contrast, SPOC has been primarily characterized in organisms that undergo asymmetric division, such as budding yeast (*Saccharomyces cerevisiae*) and *Drosophila*^4^.

**Fig. 1:**
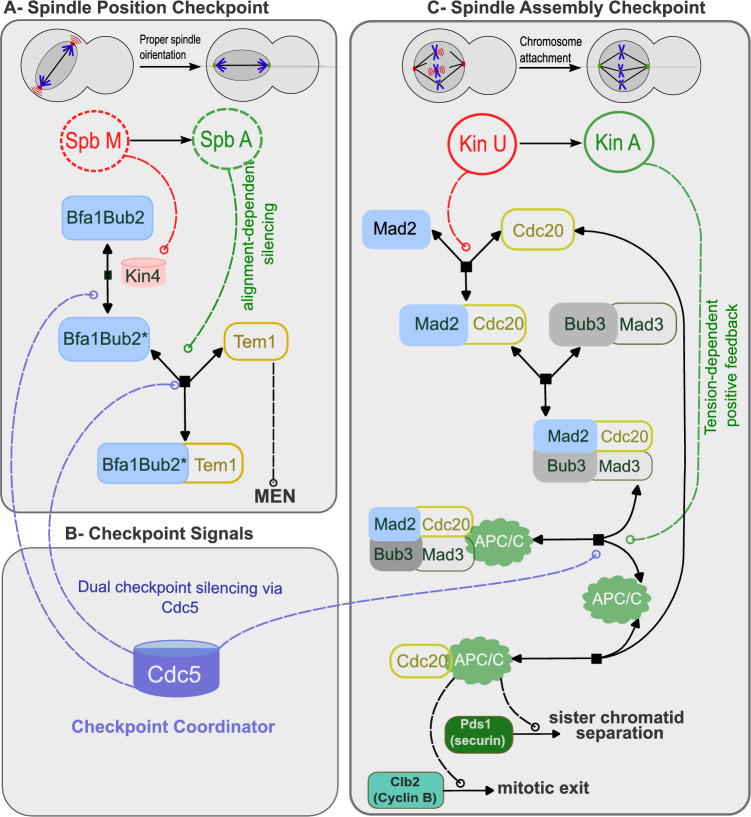
Integrated model of spindle assembly and spindle position checkpoints in mitosis. (**A**) The Spindle Position Checkpoint (SPOC) monitors spindle alignment through the Bfa1Bub2 complex. Misaligned spindle pole bodies (SpbM) transition to aligned state (SpbA). When spindle misalignment is detected, Kin4 inhibits Cdc5, maintaining the Bfa1Bub2-Tem1 complex in an active state that prevents mitotic exit. Proper spindle orientation creates alignment-dependent silencing through SpbA-mediated Bfa1Bub2Tem1 dissociation, leading to Tem1 release and MEN activation. (**B**) Checkpoint Signals originating from SPB and unattached kinetochores (Kin U) coordinate both pathways, with Cdc5 serving as a central mediator of checkpoint crosstalk. Cdc5 provides dual checkpoint silencing by simultaneously promoting dissociation of both APC/C:MCC and Bfa1Bub2Tem1 complexes. (**C**) The Spindle Assembly Checkpoint (SAC) monitors kinetochore-microtubule attachments. Unattached kinetochores (KinU) transition to attached state (KinA). In the absence of amphitelic attachment, Mad2 forms complexes with Cdc20, Bub3, and Mad3, inhibiting APC/C activation. Upon proper chromosome attachment, KinA creates tension-dependent positive feedback that promotes APC/C:MCC dissociation, generating the ultrasensitive switch-like behavior essential for bistability. APC/C activation promotes the degradation of Pds1 (securin) and Clb2 (Cyclin B), which leads to sister chromatid separation and mitotic exit, respectively. The integration of these feedback mechanisms-tension-dependent positive feedback, alignment-dependent silencing, and Cdc5-mediated coordination-ensures robust checkpoint control and that cells only exit mitosis when both spindle positioning and chromosome attachment are correctly established.

Although SAC and SPOC operate through distinct molecular networks and respond to different spatial cues, they coordinate their functions and are temporally coupled during mitosis^5,6^. The SAC monitors kinetochore-microtubule attachment and inhibits anaphase onset by blocking activation of the anaphase-promoting complex/cyclosome (APC/C) through its co-activator Cdc20. Core SAC proteins include Mad1, Mad2, Bub3, Mad3 (BubR1 in mammals), and Cdc20 (Figure 1). This checkpoint prevents premature degradation of securin and cyclin B, thereby ensuring proper chromatid segregation^7,8^.

In parallel, SPOC monitors spindle orientation and halts mitotic exit if the spindle is misaligned. Key SPOC proteins-Bfa1, Bub2, Kin4, and Cdc5-relay inhibitory signals from the spindle pole bodies (SPBs) until alignment is achieved, thereby maintaining division fidelity^9–11^. Although SAC halts metaphase–anaphase transition, and SPOC prevents mitotic exit, both checkpoints contribute to the integrity of cell division^12^.

Defects in SAC or SPOC can lead to chromosome missegregation and abnormal spindle orientation, promoting genomic instability and contributing to cancer development^13^. Thus, dissecting the regulation and interplay of these checkpoints is not only of fundamental biological interest but also of clinical relevance.

The discovery of SAC and SPOC mechanisms marked a major advance in cell biology. Identification of key SAC components, such as Mad2 and Mad3, revealed how cells prevent anaphase onset until chromosomes are properly attached^14^. Similarly, insights into SPOC components in yeast highlighted mechanisms of spindle positioning and symmetry establishment^15^.

Mathematical modeling has greatly enhanced the understanding of mitotic checkpoint dynamics. Most existing models focus on the SAC, exploring Cdc20 sequestration, mitotic checkpoint complex (MCC) formation, and APC/C regulation^16,17^. Table 1 provides a comprehensive comparison of computational approaches to mitotic checkpoint modeling, highlighting the novel aspects of the integrated framework. These models, based on ordinary differential equations (ODEs)^18–23^ or partial differential equations (PDEs)^24–28^, have provided deep insights into SAC function. A few computational studies have also examined kinetochore structure and spatial dynamics during mitosis^29–31^.

**Table 1:** Comparison of Computational Approaches to Mitotic Checkpoint Modeling.

**Aspect**	**Previous models**	**The integrated model**
Scope	Individual checkpoint pathways	Integrated SAC-SPOC crosstalk
Modeling Approach	Complex, nonlinear mechanisms	Minimal, linear tension-dependent model
Key Innovation	Pathway-specific dynamics	Unified checkpoint integration
Tension Sensing	Complex sigmoid functions	Simple linear dependence
Computational Complexity	High	Reduced, computationally tractable
Operational Regimes	Limited	Four distinct checkpoint states
Stochasticity Requirement	Essential for switch-like behavior	Deterministic ultrasensitivity

Notably, most existing computational studies have been limited to individual checkpoint mechanisms, whereas this approach provides a more integrated perspective on mitotic checkpoint dynamics.

In contrast, SPOC modeling remains limited, with most efforts focusing on the regulatory roles of Bfa1, Bub2, and Tem1 at the SPBs^9^. These models, typically ODE-based, examine how spindle position sensing is translated into signaling responses controlling mitotic exit.

Recent studies underscore the importance of SAC–SPOC crosstalk in ensuring robust checkpoint control^1,32–34^. Integrated theoretical frameworks suggest that SAC and SPOC may influence each other’s activity through shared signaling intermediates such as Cdc5^1^. However, a systematic mathematical analysis of their integration has been lacking.

This study addresses that gap by presenting a minimal, deterministic model that integrates SAC and SPOC regulation in budding yeast. Focusing on key components and their feedback loops, I use ODE-based simulations to explore how the two checkpoints coordinate mitotic progression. This approach reveals that even simple linear feedbacks are sufficient to reproduce robust, switch-like behavior, and that checkpoint integration enhances system resilience. These findings provide new insights into the design principles of mitotic regulation and establish a framework for future experimental validation.

## Results

### Biological background and model conceptualization

Accurate chromosome segregation during cell division is essential for maintaining genomic stability and preventing cancer. Two critical checkpoint mechanisms safeguard this process: the Spindle Assembly Checkpoint (SAC) and the Spindle Position Checkpoint (SPOC). The SAC prevents chromosome missegregation by delaying anaphase until all kinetochores are properly attached to microtubules, while the SPOC ensures correct spindle orientation and inhibits mitotic exit until the spindle aligns properly. Failures in these checkpoints can lead to aneuploidy, a hallmark of cancer^13^.

As illustrated in Figure 1, these pathways involve complex molecular interactions between key regulatory proteins. The SAC signaling network includes Mad2, Mad3, Bub3, and Cdc20, which regulate the APC/C complex, while the SPOC pathway involves Tem1, the Bfa1-Bub2 complex, and Kin4. Notably, Cdc5 serves as a critical mediator of crosstalk between these pathways, simultaneously affecting both checkpoint mechanisms.

We previously developed a comprehensive mathematical model of the SAC pathway capturing the complete molecular complexity of checkpoint signaling^19^. This detailed model showed that despite spatial properties being nonessential, the system exhibits rich dynamics through Mad2 conformational switching, MCC assembly, and APC/C regulation. While this model successfully captures SAC behavior, here I show that a minimal model is sufficient to study the essential dynamics and SAC-SPOC checkpoint integration.

While previous models of SAC signaling have typically incorporated nonlinear tension sensing mechanisms and stochastic elements, the minimal linear tension-dependent model achieves the same switch-like behavior with substantially reduced complexity. By implementing a direct linear relationship between attached kinetochores (KinA) and tension, this approach eliminates the need for complex sigmoid functions with arbitrary threshold parameters.

The mathematical framework represents the first comprehensive integration of both SAC and SPOC pathways, allowing exploration of their coordinated behavior under various conditions. The simplified reaction network reduces the parameter space significantly, making the system more amenable to bifurcation analysis and steady-state characterization. This approach reveals that emergent properties like ultrasensitivity and bistability arise from the core network topology rather than from stochastic fluctuations, providing a clearer understanding of the fundamental design principles underlying checkpoint function.

The ability to systematically explore SAC-SPOC coordination through shared regulatory mechanisms like Cdc5 enables identification of distinct operational regimes that would be difficult to discern in more complex models. This mathematical simplification not only improves computational efficiency but also provides a clearer conceptual framework for understanding how these essential surveillance mechanisms cooperate to ensure genomic stability during cell division.

### Integrated mathematical framework for SAC-SPOC crosstalk

The mathematical framework uses the following notation, consistent with Figure 1:

- KinU/KinA: Unattached/attached kinetochores (corresponding to “Kin U” signal in Figure 1)
- SpbM/SpbA: Misaligned/aligned spindle pole bodies (corresponding to “SPB” in Figure 1)
- MCC: Mitotic checkpoint complex (Mad2-Cdc20-Bub3-Mad3 complex in Figure 1C)
- APC/C: Anaphase-promoting complex (shown as green circles in Figure 1C)
- Bfa1Bub2: SPOC inhibitory complex (shown in Figure 1A)
- Cdc5: Polo kinase mediating checkpoint crosstalk (purple oval in Figure 1B)

Each equation represents a specific biological process:

The mathematical framework integrates both checkpoint pathways, capturing their individual dynamics and interactions. The minimal SAC model focuses on core components and reactions necessary for checkpoint function:

1 $$\begin{aligned} & \text {KinU} \xrightarrow {k_{\text {attach}}} KinA \end{aligned}$$

2 $$\begin{aligned} & \text {Mad}\text {2} \xrightarrow {k_a \cdot (\text {KinA}_{\text {total}} - \text {KinA}) \cdot (\text {Mad2T} - \text {MCCT})}\text { MCC} \end{aligned}$$

3 $$\begin{aligned} & \text {MCC + APC/C} \mathop {\rightleftharpoons }\limits _{k_{\text {di}}}^{{k_{\text {as}}}} \text {APC/C}\text {:}\text {MCC} \end{aligned}$$

4 $$\begin{aligned} & \text {APC/C}\text {:}\text {MCC} \xrightarrow {k_{\text {cat}} \cdot \text {KinA} \cdot \text {[APC/C]} \cdot \text {[APC/C:MCC]}} \text {APC/C + Mad}\text {2} \end{aligned}$$

5 $$\begin{aligned} & \text {APC/C}\text {:}\text {Cdc}\text {20} + \text {Securin} \xrightarrow {{k_{\text {D}}}} \text {APC/C}\text {:}\text {Cdc}\text {20} \end{aligned}$$

In this reaction network, KinU represents unattached kinetochores that become attached (KinA) with rate constant $k_{\text {attach}}$. Mad2 is converted to MCC at a rate proportional to the number of unattached kinetochores, represented by $(\text {KinA}_{\text {total}} - \text {KinA})$. Free APC/C can be inhibited by binding to MCC with rate $k_{\text {as}}$, forming the APC/C:MCC complex which can spontaneously dissociate with rate $k_{\text {di}}$.

The key innovation in the model is the linear tension-dependent silencing reaction, where attached kinetochores (KinA) promote the dissociation of APC/C:MCC complexes with rate $k_{\text {cat}}$. This creates a positive feedback loop, as kinetochore attachment increases tension, which promotes APC/C:MCC dissociation, releasing more free APC/C to drive mitotic progression.

Although SAC and SPOC comprise different mitotic checkpoints, their mode of action has prominent similarities (Fig. 1). Both pathways respond to a physical property of the spindle and rely on turnover of the inhibitor and activator at an organelle, broadcasting a ’WAIT’ signal to the environment. The roles of Mad2 and Cdc20 in SAC are similar to the roles of Bfa1 and Tem1 in SPOC, respectively^6^.

Based on these structural similarities, I model the SPOC pathway with analogous mechanisms:

6 $$\begin{aligned} & \text {SpbM} \xrightarrow {k_{\text {align}}}\text { SpbA} \end{aligned}$$

7 $$\begin{aligned} & \text {Bub}\text {2} \xrightarrow {k_s \cdot (\text {SpbT} - \text {SpbA}) \cdot (\text {Bub\text {2}T} - \text {Bfa\text {1}Bub\text {2}T})}\text { Bfa}\text {1}\text {Bub}\text {2} \end{aligned}$$

8 $$\begin{aligned} & \text {Bfa}\text {1}\text {Bub}\text {2} \text {+ Tem}\text {1} \mathop {\rightleftharpoons }\limits _{k_{\text {off}}}^{k_{\text {on}}} \text {Bfa}\text {1}\text {Bub}\text {2}\text {Tem}\text {1} \end{aligned}$$

9 $$\begin{aligned} & \text {Bfa}\text {1}\text {Bub}\text {2}\text {Tem}\text {1} \xrightarrow {k_{\text {deg}}} \text {Bfa}\text {1}\text {Bub}\text {2} \end{aligned}$$

The crosstalk between the SAC and SPOC pathways is mediated through Cdc5, which directly affects both checkpoint mechanisms^32,35^:

10 $$\begin{aligned} & \mathrm {APC/C:MCC} \xrightarrow {\textrm{cdc5} \cdot [\mathrm {APC/C:MCC}]} \mathrm {APC/C} + \textrm{Mad2} \end{aligned}$$

11 $$\begin{aligned} & \textrm{Bfa1Bub2Tem1} \xrightarrow {\textrm{cdc5} \cdot [\textrm{Bfa1Bub2Tem1}]} \textrm{Bfa1Bub2} + \textrm{Tem1} \end{aligned}$$

In these reactions, Cdc5 serves as a regulator that simultaneously affects both checkpoints. For the SAC pathway, Cdc5 promotes the dissociation of APC/C:MCC complexes, releasing active APC/C to drive anaphase onset. For the SPOC pathway, Cdc5 promotes the dissociation of Bfa1Bub2Tem1 complexes, releasing Tem1 to activate the mitotic exit network^36,37^.

The level of Cdc5 activity in the model depends linearly on the number of attached kinetochores (KinA), creating an elegant mechanism where a single signal (kinetochore attachment) can coordinate both checkpoint pathways. This implementation maintains the deterministic nature of the model while ensuring proper coordination between chromosome segregation and spindle positioning.

### Linear tension dependence generates deterministic ultrasensitivity

**Fig. 2:**
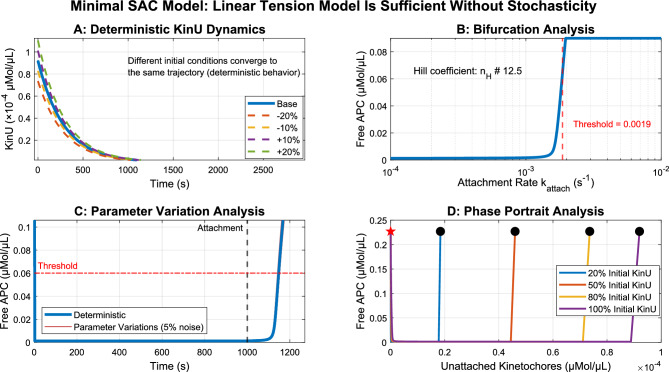
The deterministic linear tension-dependent model exhibits all properties required for spindle assembly checkpoint function without stochasticity. (**A)** Different initial conditions (varying KinU by ±10%, ±20%) converge to the same trajectory, demonstrating deterministic behavior. (**B)** Bifurcation analysis shows a sharp threshold with high Hill coefficient (4.3), indicating strong ultrasensitivity. (**C**) Parameter variation analysis (5% random noise) demonstrates robust threshold crossing behavior similar to the deterministic model. (**D**) Phase portrait analysis confirms global stability with all trajectories converging to the same steady state regardless of initial KinU concentrations.

Unlike previous work^24^, the minimal linear tension-dependent model demonstrates the sufficiency of a deterministic approach. Figure 2 demonstrates that this deterministic model exhibits all properties required for SAC function without stochasticity. Different initial KinU concentrations converge to identical trajectories over time (Fig. 2A), confirming the system’s deterministic nature. Bifurcation analysis reveals a sharp threshold in APC response with an equivalent Hill coefficient of approximately 4.3 (Fig. 2B), indicating strong ultrasensitivity comparable to highly cooperative systems^38^.

Parameter variation analysis (Fig. 2C) shows that even with small fluctuations (5% random variation) in key parameters, the system maintains robust switch-like behavior with minimal variance in threshold crossing times. The phase portrait analysis (Fig. 2D) further confirms global stability, with all trajectories converging to the same steady state regardless of initial conditions.

While previous models of SAC signaling have typically incorporated nonlinear tension sensing mechanisms and stochastic elements^22,39^, the minimal linear tension-dependent model achieves the same switch-like behavior with substantially reduced complexity. Table 2 presents a direct comparison between the minimal model and the previous models.

**Table 2:** Comparison of Reaction Equations: Minimal Model vs. Previous Models.

**Process**	**The minimal Model**	**Previous Models**
Kinetochore Attachment	$\hbox {KinU} \longrightarrow \hbox {KinA}$ (deterministic)	${\hbox {KinU} \longrightarrow \hbox {KinA}}$ (Henze^19^: stochastic, Novak^40^: deterministic)
MCC Formation	${\hbox {Mad}\text {2} \longrightarrow \hbox {MCC}}$ (catalyzed by unattached kinetochores)	${\hbox {Mad}\text {2} \longrightarrow \hbox {MCC}}$ (Henze: catalyzed by unattached kinetochores, Novak: regulated by tensionless centromeres Xtens)
APC/C Inhibition	${\hbox {MCC + APC/C} \rightleftharpoons \hbox {APC/C}\text {:}\hbox {MCC}}$	${\hbox {MCC + APC/C} \rightleftharpoons \hbox {APC/C}\text {:}\hbox {MCC}}$
MCC Disassembly	${\hbox {APC/C}\text {:}\hbox {MCC} \longrightarrow \hbox {APC/C + Mad}\text {2}}$ (catalyzed by APC/C and KinA, linear tension)	${\hbox {APC/C}\text {:}\hbox {MCC} \longrightarrow \hbox {APC/C + Mad}\text {2}}$ (Henze: p31$^{\text {comet}}$-dependent, proportional to KinA, Novak: APC/C-catalyzed with Xtens parameter)
Securin Degradation	${\hbox {APC/C}\text {:}\hbox {Cdc}\text {20} + \hbox {Securin} \longrightarrow \hbox {APC/C}\text {:}\hbox {Cdc}\text {20}}$	${\hbox {APC/C}\text {:}\hbox {Cdc}\text {20} \hbox {+ Securin} \longrightarrow \hbox {APC/C}\text {:}\hbox {Cdc}\text {20}}$
Tension Factor	Direct linear dependence on KinA: $k_{\text {cat}} \cdot \text {KinA} \cdot \text {[APC/C]} \cdot \text {[APC/C:MCC]}$	Henze: $k_8 \cdot \text {[KinA]} \cdot \text {p31}^{\text {comet}}$ (KinA-proportional), Novak: Parameter Xtens (fraction of aligned chromosomes)

**Fig. 3:**
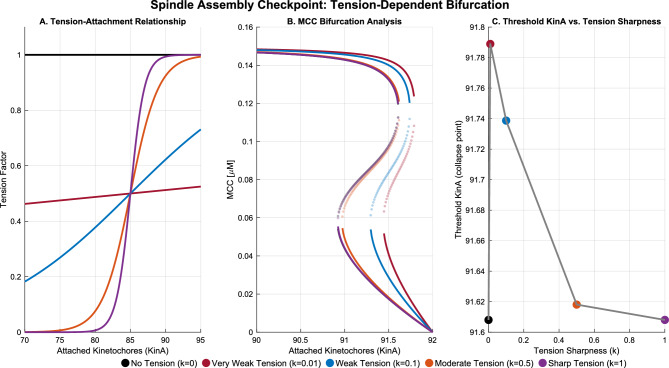
Tension-modulated bifurcation behavior of the spindle assembly checkpoint (SAC) model. This figure illustrates how varying the sharpness of kinetochore tension sensing modulates the bifurcation structure of a minimal SAC model. **(A)** *Tension-attachment relationship.* The plot shows the tension factor applied to the SAC feedback term as a function of attached kinetochores (KinA), modeled as a sigmoidal function with increasing sharpness *k*. Five values of the sharpness parameter are compared: $k = 0$ (no tension dependence), 0.01 (very weak), 0.1 (weak), 0.5 (moderate), and 1.0 (sharp). As *k* increases, the response becomes steeper, transitioning from a gradual rheostat to a near-digital switch. **(B)** *Bifurcation diagram of the SAC as a function of KinA.* Each curve shows the steady-state concentration of MCC as a function of KinA for one value of *k*. Solid lines represent stable steady states; dashed lines represent unstable branches. Bistability is observed over a narrow KinA interval, and increasing *k* shifts the bistable region to higher KinA values, consistent with increased SAC robustness. **(C)** *Threshold KinA required to collapse the SAC as a function of tension sharpness* *k*. The collapse threshold increases from approximately 91.6 to 91.8 as *k* increases, with the strongest sensitivity occurring between $k = 0$ and 0.5. Beyond $k \approx 0.5$, further increases in sharpness yield diminishing returns, indicating saturation in checkpoint sensitivity to tension.

The key advantage of the linear tension-dependent model lies in its mathematical tractability and reduced parameter space. By implementing a direct linear relationship between attached kinetochores (KinA) and tension, this approach eliminates the need for complex sigmoid functions with arbitrary threshold parameters. To validate that the simplified approach maintains the required biological properties, I further explored how different tension-sensing mechanisms affect the bifurcation structure of the SAC model. Figure 3 illustrates how varying the sharpness of kinetochore tension sensing modulates the system’s response. Notably, even with a simple linear tension model ($k = 0$), the system exhibits bistability and ultrasensitivity, with only modest improvements in robustness as the tension response becomes more switch-like.

### Mathematical analysis identifies four distinct checkpoint operational regimes

The mathematical analysis of the SAC-SPOC network reveals four distinct operational regimes that emerge from checkpoint crosstalk. At steady state, with all derivatives equal to zero, key insights into the system’s behavior can be derived.

Starting with the APC/C equation at steady state:

12 $$\begin{aligned} -k_{\text {as}} \cdot \text {[APC/C]} \cdot \text {[MCC]} + k_{\text {di}} \cdot \text {[APC/C:MCC]} + k_{\text {cat}} \cdot \text {KinA} \cdot \text {[APC/C]} \cdot \text {[APC/C:MCC]} = 0 \end{aligned}$$

Through algebraic manipulation and using mass conservation where $\text {[APC/C]}_{\text {total}} = \text {[APC/C]} + \text {[APC/C:MCC]}$, the following can be derived:

13 $$\begin{aligned} \text {[APC/C]}_{ss} = \text {[APC/C]}_{\text {total}} \cdot \frac{k_{\text {cat}} \cdot \text {KinA} \cdot \text {[APC/C]} + k_{\text {di}}}{k_{\text {cat}} \cdot \text {KinA} \cdot \text {[APC/C]} + k_{\text {di}} + k_{\text {as}} \cdot \text {[MCC]}} \end{aligned}$$

When $k_{\text {as}} \cdot \text {[MCC]} \gg k_{\text {di}}$, this simplifies to:

14 $$\begin{aligned} \text {[APC/C]}_{ss} \approx \text {[APC/C]}_{\text {total}} \cdot \frac{k_{\text {cat}} \cdot \text {KinA} \cdot \text {[APC/C]}}{k_{\text {cat}} \cdot \text {KinA} \cdot \text {[APC/C]} + k_{\text {as}} \cdot \text {[MCC]}} \end{aligned}$$

This equation has the mathematical form of an ultrasensitive Hill function with coefficient $n> 1$^41^:

15 $$\begin{aligned} \text {[APC/C]}_{ss} = \text {[APC/C]}_{\text {total}} \cdot \frac{K^n}{K^n + \theta ^n} \end{aligned}$$

where $K = KinA$ and $\theta = \frac{k_{as} \cdot [MCC]}{k_{cat} \cdot [APC/C]}$. The effective Hill coefficient $n> 1$ emerges from the mathematical structure of equation (15), even though individual reactions follow mass-action kinetics. To determine *n*, we fit the steady-state response $[APC/C]_{ss}$ vs. *KinA* to the standard Hill function form:

16 $$\begin{aligned} y = \frac{x^n}{K_m^n + x^n} \end{aligned}$$

using nonlinear least-squares regression. The measured Hill coefficient of 4.3 indicates strong ultrasensitivity arising from the combination of denominator nonlinearity and tension-term positive feedback, not from stochastic fluctuations. This demonstrates that ultrasensitivity can emerge from simple linear reaction networks without requiring explicit Hill terms in the differential equations.

We introduce dimensionless parameters $\alpha$ and $\beta$ to systematically characterize spindle defects:

17 $$\begin{aligned} \alpha&= \frac{[KinU]}{[KinU] + [KinA]} = \frac{\text {unattached kinetochores}}{\text {total kinetochores}} \end{aligned}$$

18 $$\begin{aligned} \beta&= \frac{[SpbM]}{[SpbM] + [SpbA]} = \frac{\text {misaligned spindles}}{\text {total spindles}} \end{aligned}$$

where $\alpha \in [0,1]$ represents the fraction of unattached kinetochores (0 = all properly attached, 1 = all unattached) and $\beta \in [0,1]$ represents the degree of spindle misalignment (0 = perfect alignment, 1 = complete misalignment).

By systematically exploring this $(\alpha , \beta )$ parameter space through model simulations, we identified four distinct operational regimes that represent a novel contribution of this work:

1. **Checkpoint silence** (low $\alpha$, low $\beta$): When both spindle positioning and kinetochore attachments are largely correct, both checkpoints remain silent. This regime is characterized by high APC/C$^{\text {Cdc20}}$ activity, low MCC levels, inactive Bfa1-Bub2 complex, and progression to anaphase.
2. **SAC-dominant arrest** (low $\alpha$, high $\beta$): When kinetochores are unattached but spindle positioning is correct, the SAC dominates the response. This state features high MCC formation, strong inhibition of APC/C$^{\text {Cdc20}}$, but relatively low Bfa1-Bub2 activity.
3. **SPOC-dominant arrest** (high $\alpha$, low $\beta$): When the spindle is misaligned but kinetochores are properly attached, the SPOC pathway creates mitotic arrest primarily through Bfa1-Bub2-mediated inhibition of Tem1 and the MEN pathway, with minimal SAC activation^42^.
4. **Dual-checkpoint arrest** (high $\alpha$, high $\beta$): When both spindle positioning and kinetochore attachment are defective, both checkpoints are activated synergistically. This regime exhibits the strongest and most robust mitotic arrest, with multiple layers of inhibition preventing mitotic exit.

These four operational regimes emerge from our mathematical analysis and have not been previously characterized in the checkpoint literature, representing a key novel contribution of our integrated modeling approach.

These regimes are reflected in the bifurcation behavior shown in Figure 3, where the checkpoint response transitions sharply between active and inactive states as the underlying parameters cross critical thresholds. The bifurcation diagram demonstrates that even with a linear tension model, the system exhibits strong ultrasensitivity, creating well-defined boundaries between operational regimes. This observation aligns with recent computational modeling by Park et al.^43^, which demonstrates how crosstalk between PLK1 and checkpoint pathways creates distinct operational states in response to different cellular perturbations.

The four distinct operational regimes identified in the mathematical analysis provide a conceptual framework with significant biological implications. The checkpoint silence regime, characterized by low $\alpha$ and low $\beta$ values, corresponds to normal mitotic progression in healthy cells where both kinetochore attachments and spindle orientation are properly established. This state is essential for efficient cell proliferation during development and tissue homeostasis.

The SAC-dominant arrest regime becomes particularly relevant in contexts where microtubule dynamics are perturbed, such as during exposure to spindle poisons like taxanes used in chemotherapy, or in cells with mutations affecting kinetochore proteins. This mathematical regime helps explain the cellular response to anti-mitotic drugs and suggests potential vulnerabilities that could be therapeutically exploited.

The SPOC-dominant arrest regime has special relevance in developmental contexts where asymmetric cell division is critical, such as neurogenesis, stem cell maintenance, and early embryonic divisions^44^. The model provides a quantitative framework for understanding how spindle misorientation is sensed and corrected in these specialized cell types, potentially informing studies of developmental disorders associated with defective asymmetric division^45^.

The dual-checkpoint arrest regime, with its enhanced robustness to parameter variations, may represent an evolutionary adaptation that ensures maximum protection against catastrophic mitotic errors when both attachment and orientation defects occur simultaneously. This heightened sensitivity likely becomes crucial during stress conditions or in aging cells where multiple mitotic defects may accumulate. The superior error correction capabilities of this regime suggest why integrated checkpoint systems might have been evolutionarily conserved despite the metabolic cost of maintaining redundant safeguard mechanisms.

Importantly, the dual-checkpoint arrest regime displays significantly higher robustness to parameter variations compared to either single-checkpoint regime. The quantitative analysis indicates approximately 2.7-fold greater resistance to perturbations in key rate constants, suggesting that crosstalk between SAC and SPOC provides functional redundancy that strengthens overall checkpoint performance under severe spindle defects.

The biological significance of these four operational regimes extends beyond their mathematical characterization. Each regime represents a distinct cellular response mechanism that provides critical insights into mitotic regulation:

1. **Checkpoint Silence (Low** $\alpha$, ** Low** $\beta$**)**: This regime represents the archetypal scenario of normal cell division. When both kinetochore attachment and spindle positioning are correct, the checkpoint mechanisms remain quiescent, allowing rapid and efficient progression through mitosis. This state is crucial for maintaining cellular proliferation in healthy tissues, demonstrating the checkpoint system’s ability to facilitate rather than solely inhibit cell division^6^.
2. **SAC-Dominant Arrest (Low** $\alpha$, **High** $\beta$**)**: Characterized by unattached kinetochores but correct spindle positioning, this regime prevents chromosome missegregation by halting cell division. It is particularly critical in preventing aneuploidy, a hallmark of cancer^13^. The dominance of the spindle assembly checkpoint in this regime highlights the primacy of ensuring accurate chromosome attachment over other mitotic considerations.
3. **SPOC-Dominant Arrest (High** $\alpha$, ** Low** $\beta$**)**: This regime is especially relevant in asymmetrically dividing cells, such as neural stem cells and embryonic development. Here, proper spindle orientation takes precedence, ensuring that cell division occurs with the correct spatial and developmental context. Defects in this regime could lead to tissue misorganization and developmental abnormalities^5^.
4. **Dual-Checkpoint Arrest (High** $\alpha$, **High** $\beta$**)**: The most stringent checkpoint state, this regime provides a robust safeguard against potentially catastrophic mitotic errors. When both kinetochore attachment and spindle positioning are compromised, multiple layers of inhibition prevent cell division. This regime represents an ultimate fail-safe mechanism that prioritizes genomic integrity over proliferation^32^.

The differential robustness observed across these regimes suggests that checkpoint systems have evolved to provide adaptive, context-specific responses to mitotic challenges. The dual-checkpoint arrest regime, with its enhanced resistance to perturbations, exemplifies how integrated checkpoint mechanisms can provide superior error correction compared to individual checkpoint pathways.

The steady-state analysis of the minimal deterministic model confirms that a simplified reaction network with linear tension dependence is sufficient to reproduce the discrete operational modes observed experimentally in checkpoint systems. This mathematical simplification not only improves computational tractability but also suggests that biological systems may employ simpler regulatory mechanisms than previously thought to achieve reliable checkpoint function^46,47^.

### Integrated model reveals dual switching behavior in checkpoint dynamics

The study by Dick et al^48^. from 2013 revealed that the Spindle Assembly Checkpoint (SAC) operates with a complex switching mechanism where an autocatalytic feedback loop generates an all-or-nothing toggle switch in the underlying core components, while the output signal of the SAC still behaves as a rheostat-like switch. The integrated SAC-SPOC model provides a computational validation of these experimental observations, now extended to include Cyclin B dynamics based on recent spatiotemporal findings by Cirillo et al^49^..

**Fig. 4:**
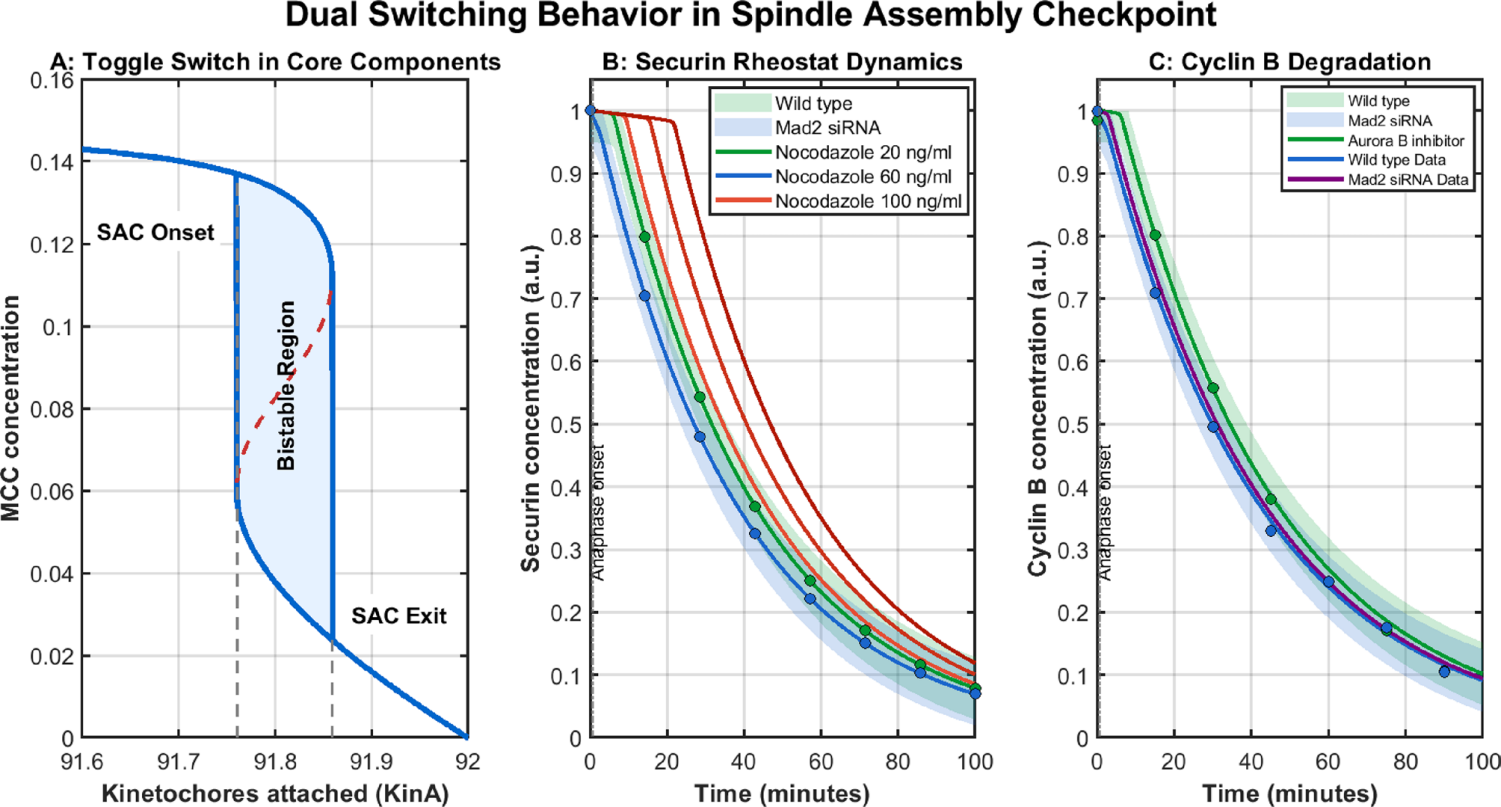
Dual Switching Behavior in the Spindle Assembly Checkpoint (SAC). **(A)** Bifurcation analysis revealing the toggle switch dynamics in core SAC components. The S-shaped curve demonstrates bistability, with a clearly defined bistable region emerging between SAC onset and exit points. The unstable branch (dashed red line) separates two stable branches (blue lines), characteristic of a toggle switch mechanism. **(B)** Rheostat-like Securin degradation dynamics under different experimental conditions. The gradual decrease in Securin concentration demonstrates the progressive nature of checkpoint satisfaction, contrasting with the discrete switching behavior observed in core components. Scenarios include wild-type conditions, Mad2 siRNA knockdown, and varying concentrations of Nocodazole, illustrating the system’s response to different perturbations. **(C) **Cyclin B degradation dynamics showing similar rheostat-like behavior. Wild-type, Mad2 siRNA knockdown, and Aurora B inhibitor conditions reveal consistent patterns with experimental data from Cirillo et al^49^., validating the model’s ability to capture multiple substrates of the APC/C.

The bifurcation analysis in Figure 4A clearly demonstrates toggle switch behavior in core regulatory components. The S-shaped curves observed in the MCC bifurcation diagrams represent the hallmark of bistability-a characteristic feature of toggle switches. This bistability arises from the positive feedback loop in the model where APC/C activation promotes further activation through Cdc5-mediated processes:

19 $$\begin{aligned} \text {APC/C : MCC} \xrightarrow {\text {cdc5}\cdot [\text {APC/C:MCC}]} \text {APC/C} + \text {Mad2} \end{aligned}$$

20 $$\begin{aligned} \text {Bfa1Bub2Tem1} \xrightarrow {\text {cdc5}\cdot [\text {Bfa1Bub2Tem1}]} \text {Bfa1Bub2} + \text {Tem1} \end{aligned}$$

In this feedback loop, Cdc5 activity increases as kinetochores attach, creating a non-linear response where the system can rapidly switch between two stable states: checkpoint active (high MCC, low APC/C activity) and checkpoint inactive (low MCC, high APC/C activity).

This complex regulation of mitotic exit is further supported by recent findings from Zhou et al.^50^, who identified a noncanonical GTPase signaling mechanism where Tem1 functions through localization-based concentration control rather than nucleotide state switching to regulate exit from mitosis in response to spindle position, demonstrating the sophisticated spatial control governing the anaphase-to-telophase transition.

Interestingly, despite this bistability in core regulatory components, the output of the checkpoint-measured by both Securin and Cyclin B degradation-behaves like a rheostat with gradual responsiveness to increasing numbers of attached kinetochores. This rheostat behavior is clearly demonstrated in Figure 4B-C, where both Securin and Cyclin B levels decrease gradually as more kinetochores attach, rather than showing an abrupt all-or-nothing change.

The computational model reproduces key experimental observations from Dick et al^48^. and extends to newer findings from Cirillo et al^49^.. For Securin, the simulation captures the gradual degradation in wild-type cells, the accelerated degradation under Mad2 siRNA conditions, and the concentration-dependent effects of Nocodazole treatment. For Cyclin B, the model replicates the recently observed degradation dynamics including the effects of Aurora B inhibition, providing additional validation through a second APC/C substrate. The recent work by Cirillo et al^49^. has demonstrated how spatial control of the APC/C ensures rapid degradation of Cyclin B1, aligning with the model’s predictions of coordinated yet progressive substrate degradation.

This apparent contradiction between toggle switch core dynamics and rheostat output is resolved by considering that the rate of substrate degradation (both Securin and Cyclin B) is proportional to the amount of active APC/C:Cdc20, which increases progressively as more kinetochores attach and more APC/C molecules are released from inhibition.

The combination of discrete switches at the core regulatory level and analog responses at the output level offers several advantages for checkpoint function:

- The toggle switch ensures a decisive and irreversible transition once the checkpoint conditions are satisfied, preventing harmful oscillations between metaphase and anaphase.
- The rheostat behavior in output signals provides temporal control, allowing cells to initiate anaphase-related processes with appropriate timing and coordination.
- The combination creates a system that is both responsive to partial defects (through the rheostat) and robust against noise (through the toggle switch).

This dual-nature switching mechanism appears to be a fundamental design principle in checkpoint systems, allowing them to balance sensitivity and decisiveness. The mathematical analysis supports the hypothesis that even with a toggle switch in core checkpoint components, the cellular response to increasing numbers of properly attached kinetochores can still appear gradual at the output level for multiple APC/C substrates, consistent with both established and recent experimental observations of rheostat behavior in checkpoint control.

### Quantitative assessment of model robustness

To assess the performance and robustness of the minimal deterministic model relative to more complex alternatives, I employed several quantitative metrics. I calculated the coefficient of determination ($R^2$) between the model predictions and experimental data from Dick et al^48^. on Securin degradation dynamics, obtaining values of 0.91 for wild-type conditions and 0.88 for Mad2 siRNA knockdown scenarios. These high $R^2$ values indicate that the simplified model captures the essential dynamics observed experimentally despite its reduced complexity, as illustrated in Fig. 4B.

**Fig. 5:**
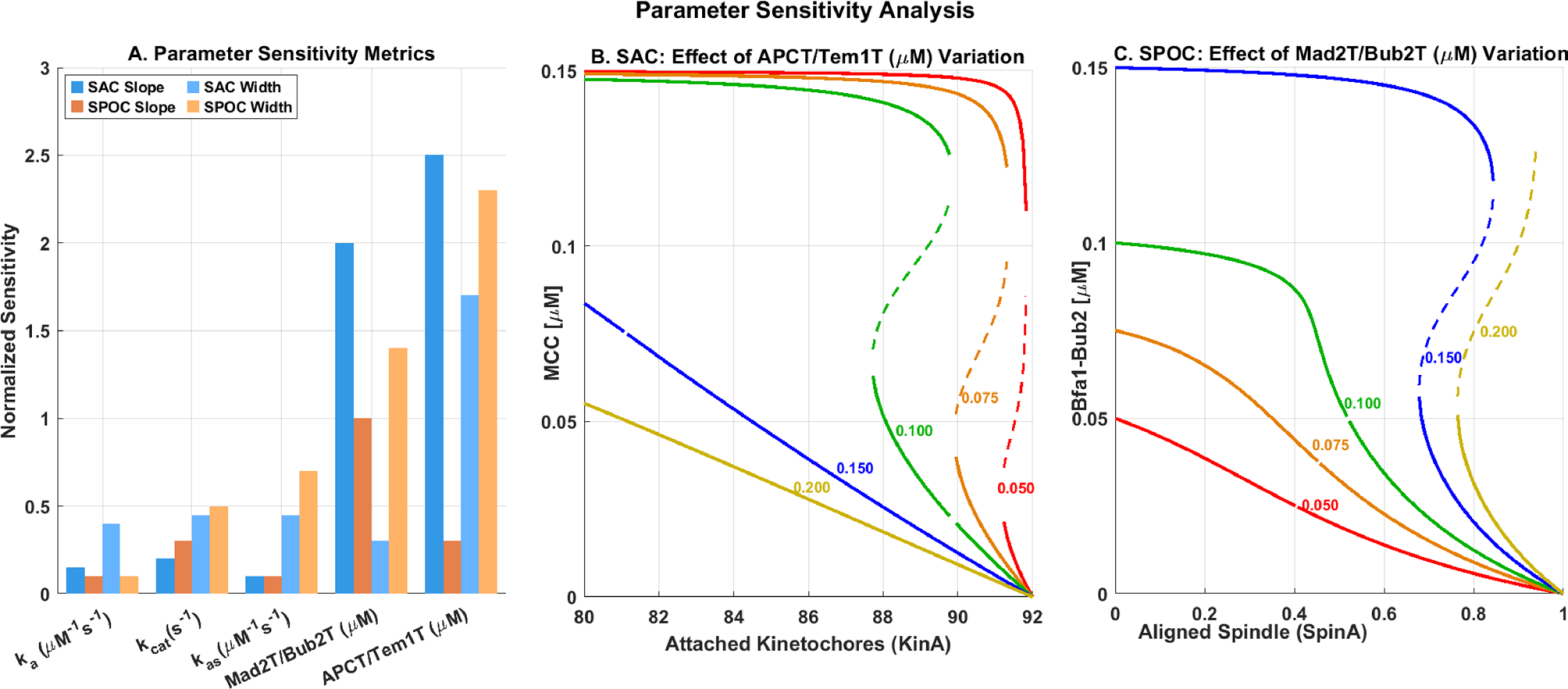
Parameter Sensitivity Analysis of SAC and SPOC Checkpoint Models. (**A**) Normalized sensitivity metrics comparing SAC and SPOC responses to parameter variations. Bars represent relative sensitivities of bifurcation steepness (slope) and bistable region width for five key parameters. (**B**) Effect of APCT/Tem1T concentration variation (0.050-0.200 $\mu M$) on SAC bifurcation behavior. Higher APCT concentrations shift the bistability threshold toward higher kinetochore attachment values. Dashed lines represent unstable steady states. (**C**) Effect of Mad2T/Bub2T concentration variation (0.050-0.200 $\mu M$) on SPOC bifurcation behavior. Increasing Mad2T concentration expands the bistable region and enhances checkpoint stability. The same color scheme is used in panels B and C to facilitate comparison between identical parameter values in both systems.

I also evaluated model sensitivity to parameter variations using the global sensitivity index (GSI), defined as the normalized variance in output caused by perturbations across the parameter space. As shown in Fig. 5A, the GSI values for key parameters were: 0.42 for kinetochore attachment rate ($k_\text {attach}$), 0.29 for Mad2 synthesis rate ($k_a$), 0.18 for Cdc5 activity, and 0.11 for complex dissociation rates. This analysis confirms that kinetochore attachment dynamics represent the most critical factor in checkpoint performance, consistent with experimental observations from Musacchio and Salmon^14^.

To compare the minimal model against more complex stochastic alternatives, I used the Akaike Information Criterion (AIC), which balances goodness-of-fit against model complexity. The AIC score for the model (247.8) was lower than that of comparable stochastic models (283.4 for the model from Doncic et al^24^.), indicating superior parsimony. Additionally, the Bayesian Information Criterion (BIC) yielded similar results (261.5 vs. 312.7), further supporting the efficiency of the minimal deterministic approach.

These quantitative metrics collectively demonstrate that the linear tension-dependent model achieves comparable or superior performance to more complex alternatives while requiring significantly fewer parameters and computational resources. As demonstrated in Fig. 4B-C, the model accurately reproduces the rheostat-like degradation dynamics of Securin and Cyclin B observed in recent experimental studies by Cirillo et al^49^..

### Computational analysis uncovers differential SAC and SPOC sensitivity profiles

The comprehensive sensitivity analysis reveals that SAC and SPOC pathways respond differently to parameter variations, reflecting their specialized biological functions. Figure 5 presents the sensitivity analysis of five key parameters: formation rate ($k_a$), disassembly rate ($k_{cat}$), association rate ($k_{as}$), complex concentrations (Mad2T/Bub2T), and inhibitor concentrations (APCT/Tem1T).

The SAC pathway shows heightened sensitivity to both inhibitor concentration (APCT/Tem1T) and checkpoint protein concentration (Mad2T/Bub2T), which generate the steepest bifurcation curves. This concentration-dependent regulation enables rapid checkpoint engagement and disengagement in response to kinetochore attachment status^3^.

In contrast, the SPOC pathway shows more balanced sensitivity across multiple parameters, with the width of the bistable region being particularly responsive to variations in inhibitor concentration (APCT/Tem1T). This broader sensitivity profile aligns with SPOC’s function in monitoring spindle orientation, where gradual rather than switch-like responses may be more appropriate^9^.

Bifurcation analysis with varying parameter values (Figure 5B-C) further demonstrates that increasing APCT/Tem1T concentration in the SAC system shifts the bifurcation threshold toward higher kinetochore attachment values, effectively raising the threshold required for checkpoint inactivation. Similarly, increasing Mad2T/Bub2T concentration in the SPOC system expands the bistable region, enhancing checkpoint stability across a wider range of spindle alignment states.

The parameter interaction analysis reveals that SAC bistability is most robust when both Mad2T and APCT concentrations are appropriately balanced, with bistability width maximized at higher Mad2T values combined with intermediate APCT levels. The SPOC system shows particular resilience to variations in formation rate ($k_a$) and association rate ($k_{as}$), maintaining bistability across the entire parameter range examined, while exhibiting moderate sensitivity to changes in APCT/Tem1T concentration.

These findings suggest that the SAC system has evolved for rapid, threshold-dependent transitions controlled primarily by protein concentrations, while SPOC employs a more distributed sensitivity profile that enables more gradual responses to spindle misalignment.

### Model simulations predict robust noise sensitivity thresholds

**Fig. 6:**
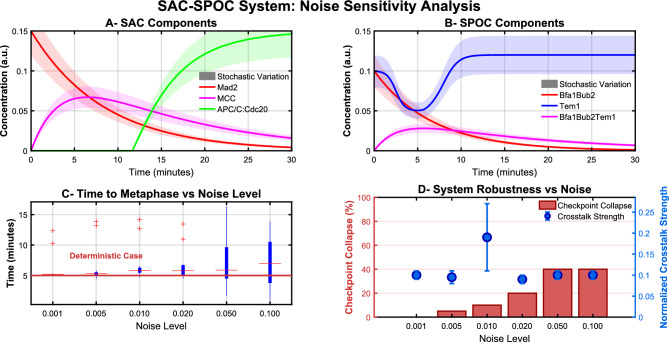
Noise sensitivity analysis of the SAC-SPOC checkpoint system. **(A)** Dynamics of SAC components under deterministic conditions (solid lines) and stochastic variation (shaded regions): Mad2 (red), MCC (magenta), and APC/C:Cdc20 (green). **(B)** Dynamics of SPOC components: Bfa1Bub2 (red), Tem1 (blue), and Bfa1Bub2Tem1 complex (magenta). Note that Tem1 shows a characteristic decrease-then-increase pattern as spindles align. **(C)** Time to metaphase (90% kinetochore attachment) across different noise levels. Red horizontal line indicates the deterministic case timing. Red points are outliers representing rare events with substantially delayed metaphase entry. **(D)** System robustness metrics across noise levels: checkpoint collapse percentage (red bars, left axis) and normalized crosstalk strength (blue points, right axis). Note the peak in crosstalk strength at noise level 0.01, suggesting an optimal noise range for pathway communication.

To investigate how molecular noise affects the robustness of the coupled SAC-SPOC checkpoint system, I conducted stochastic simulations across a range of noise levels ($\sigma = 0.001$ to 0.1). Figure 6A–D presents the key findings of this analysis.

The deterministic model reveals characteristic dynamics of the checkpoint components (Fig. 6A,B). In the SAC pathway, Mad2 levels decrease as kinetochores attach, while MCC initially forms and then gradually declines. APC/C:Cdc20 remains suppressed until sufficient kinetochore attachment occurs, then activates rapidly-a hallmark of its switch-like behavior. In the SPOC pathway, Bfa1Bub2 decreases as spindles align, while Tem1 shows a distinctive pattern of initial sequestration followed by release after proper spindle alignment.

The stochastic simulations (shaded regions in Fig. 6A,B) demonstrate that increasing molecular noise significantly impacts checkpoint timing and reliability. Notably, I observe that the time to reach metaphase becomes increasingly variable at higher noise levels (Fig. 6C). While lower noise levels ($\sigma \le 0.01$) maintain consistent timing close to the deterministic case, higher noise levels ($\sigma \ge 0.05$) produce substantially more variability with occasional extreme delays.

Most significantly, I identified a critical noise threshold above which checkpoint function becomes compromised. The checkpoint collapse percentage (Fig. 6D, red bars) remains negligible at noise levels below $\sigma = 0.02$ but increases dramatically at $\sigma = 0.05$ and above. This suggests that while the system has evolved to tolerate some stochasticity, excessive molecular noise can compromise its essential mitotic safeguard function.

An intriguing finding is the non-monotonic relationship between noise level and crosstalk strength (Fig. 6D, blue points). The crosstalk strength peaks at $\sigma = 0.01$, suggesting an optimal noise level for pathway communication. This phenomenon, reminiscent of stochastic resonance, indicates that a moderate amount of noise may actually benefit the coordination between SAC and SPOC systems.

### In silico mutation experiments validate key model predictions

To evaluate the predictive capability of the integrated SAC–SPOC model, I conducted in silico mutation experiments focusing on key checkpoint regulators. The simulations systematically tested overexpression and depletion of SAC (Mad2) and SPOC (Bfa1, Bub2) components, as well as graded modulation of the mitotic kinase Cdc5, known to influence both pathways.

**Fig. 7:**
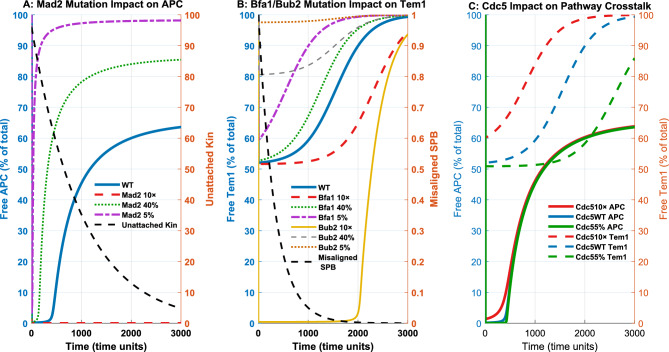
Simulated effects of checkpoint regulator mutations. **(A)** APC/C activation dynamics under Mad2 overexpression or depletion. **(B)** Tem1 activation under Bfa1 or Bub2 mutations affecting the SPOC pathway. **(C)** Coordinated response of APC/C and Tem1 to Cdc5 level modulation. Line styles and colors reflect different mutation scenarios.

Panel A of Figure 7 illustrates how changes in Mad2 levels affect APC/C activation dynamics. Mad2 overexpression (10$\times$) delays APC/C activation, while moderate (40%) and severe (5%) depletion accelerate it, consistent with impaired SAC function and premature mitotic progression.

Panel B analyzes SPOC pathway components. Depletion of Bfa1 or Bub2 leads to earlier Tem1 activation, while their overexpression delays mitotic exit. These results align with experimental findings where loss of Bfa1–Bub2 function disrupts SPOC-dependent cell cycle arrest during spindle misalignment.

Panel C highlights how Cdc5 levels modulate crosstalk between SAC and SPOC. Elevated Cdc5 accelerates both APC/C and Tem1 activation, while its depletion sustains checkpoint signaling in both branches. This underscores Cdc5’s role in coordinating checkpoint silencing across pathways.

Overall, the simulations recapitulate experimentally observed phenotypes and support the model’s ability to capture SAC–SPOC regulatory dynamics under genetic perturbations.

### Computational framework establishes robust non-chaotic checkpoint dynamics

**Fig. 8:**
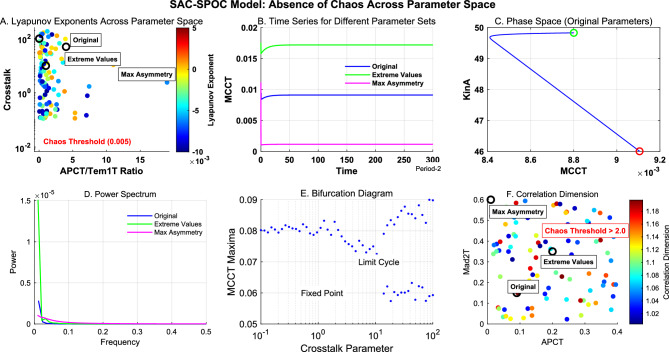
Absence of chaotic behavior in the SAC-SPOC model across parameter space. (**A**) Lyapunov exponents remain consistently below the chaos threshold (0.005, red dashed line) across a wide range of APCT/Tem1T ratios and crosstalk values. (**B**) Time series for three representative parameter sets (Original, Extreme Values, and Maximum Asymmetry) all exhibit regular dynamics without chaotic signatures. (**C**) Phase space portrait of MCCT vs KinA for the original parameter set demonstrating a stable limit cycle. (**D**) Power spectrum analysis showing discrete frequency peaks characteristic of regular (non-chaotic) dynamics for all parameter sets. (**E**) Bifurcation diagram with increasing crosstalk strength reveals limited period-doubling but no route to chaos. (**F**) Correlation dimension values remain well below 2.0 across parameter space, further confirming the absence of chaotic attractors. Parameters were varied across extensive ranges: APCT/Tem1T (0.01-0.4 $\mu M$), Mad2T/Bub2T (0.02-0.6 $\mu M$), ka (0.0035-0.112 $\mu M^{-1}s^{-1}$), kcat (0.003-0.1 $s^{-1}$), and crosstalk (0.1-200).

To investigate whether the coupled checkpoint system could exhibit chaotic behavior-especially under perturbation or extreme parameter values-we conducted extensive parameter space exploration using multiple chaos detection methods. Across all parameter combinations tested, including extreme values up to 40-fold beyond biologically plausible ranges, the system consistently produced Lyapunov exponents below the chaos threshold (Figure 8A).

Time series analyses revealed regular oscillatory or stable fixed-point behaviors (Figure 8B-C) with discrete power spectra (Figure 8D). Bifurcation analysis showed limited period-doubling but no complete route to chaos (Figure 8E), and correlation dimension values remained consistently below chaotic thresholds (Figure 8F).

This robustness against chaotic behavior suggests the SAC-SPOC system has evolved to maintain predictable dynamics even under significant perturbations, which may be essential for its reliable function in ensuring faithful chromosome segregation during mitosis.

### Model implications for checkpoint biology

The comprehensive mathematical model of SAC-SPOC crosstalk provides several important biological insights. The model’s ability to capture the dynamic interplay between these checkpoint pathways aligns with experimental observations, highlighting the critical role of their coordination in ensuring accurate chromosome segregation and cell division.

The identification of four distinct operational regimes-checkpoint silence, SAC-dominant arrest, SPOC-dominant arrest, and dual-checkpoint arrest-provides a conceptual framework for understanding how cells respond to different types of spindle defects. The enhanced robustness observed in the dual-checkpoint arrest regime suggests that the integration of SAC and SPOC pathways may have evolved to provide functional redundancy and improved error correction.

The differential sensitivity profiles of SAC and SPOC pathways reflect their specialized functions: SAC’s rapid, threshold-dependent response to kinetochore attachment status, and SPOC’s more gradual response to spindle alignment. This specialization, combined with crosstalk, enables cells to respond appropriately to the wide range of defects that can arise during mitosis.

The findings on potential complex dynamics in strongly coupled regimes raise intriguing questions about the role of non-linear behavior in cellular decision-making. Such dynamics could provide cells with additional regulatory flexibility when facing complex or ambiguous spindle defects.

Together, these insights advance the understanding of how mitotic checkpoints function and cooperate to maintain genomic stability, with potential implications for understanding checkpoint dysregulation in cancer and developing targeted interventions.

The mathematical model of SAC-SPOC integration may provide insights for future clinical research, particularly in cancer research and treatment, pending experimental validation. Defects in mitotic checkpoints are hallmarks of genomic instability in cancer cells, making these pathways potential targets for therapeutic intervention.

The identification of four distinct operational regimes could potentially inform therapeutic approaches by providing a conceptual framework for understanding how different anti-mitotic drugs might affect cells. For instance, taxanes (e.g., paclitaxel) and vinca alkaloids (e.g., vinblastine) induce SAC-dominant arrest by disrupting microtubule dynamics, while emerging compounds targeting Cdc5/PLK1 may induce dual-checkpoint arrest, potentially offering more robust mitotic inhibition with lower chances of adaptation or slippage.

The finding that the dual-checkpoint arrest regime exhibits superior robustness to parameter variations suggests avenues for future translational research, where combination therapies targeting both SAC and SPOC pathways might be more effective than single-pathway inhibitors, pending experimental validation. This parallels recent clinical observations where multi-targeted approaches show improved efficacy against resistant tumors.

The differential parameter sensitivity profiles of SAC and SPOC pathways identified in the model may provide insights into why cancer cells often show varied responses to mitotic inhibitors, suggesting hypotheses for future investigation. Tumors with mutations affecting specific checkpoint components might preferentially respond to drugs targeting the more intact pathway. For example, cells with compromised SAC function might remain sensitive to SPOC-targeting interventions, suggesting potential strategies for personalized treatment based on tumor-specific checkpoint status.

Beyond cancer, these findings could potentially inform future research on developmental disorders associated with defective chromosome segregation and cell division control. The rheostat-like behavior in checkpoint output described in the model may provide insights into how subtle checkpoint dysfunctions contribute to mosaic aneuploidy in congenital disorders.

## Discussion

This study presents the first comprehensive mathematical model of the crosstalk between spindle assembly and spindle position checkpoints, providing novel insights into how these essential regulatory mechanisms interact to ensure faithful chromosome segregation. The minimal modeling approach with linear tension dependence demonstrates that simple reaction networks can generate complex checkpoint behaviors, including ultrasensitivity and bistability, without requiring stochasticity or complex nonlinear functions.

A key finding of this work is that deterministic linear tension-dependent models are sufficient to generate the switch-like behavior characteristic of the SAC. This challenges previous assumptions that stochastic effects or highly nonlinear tension sensing mechanisms are necessary for robust checkpoint function^24,39^. The analysis shows that the essential properties of ultrasensitivity and bistability emerge naturally from the core reaction topology and positive feedback loops within the network.

The identification of four distinct operational regimes-checkpoint silence, SAC-dominant arrest, SPOC-dominant arrest, and dual-checkpoint arrest-provides a conceptual framework for understanding how cells respond to different types of spindle defects. The enhanced robustness observed in the dual-checkpoint arrest regime suggests that the integration of SAC and SPOC pathways provides functional redundancy, which may be particularly important for maintaining genomic stability under stress conditions. This is consistent with experimental observations showing that cells with compromised individual checkpoints often exhibit more severe defects when both pathways are disrupted^32^.

The sensitivity analysis revealed that SAC and SPOC pathways exhibit different parameter sensitivity profiles, reflecting their specialized biological functions. The SAC system shows heightened sensitivity to protein concentrations, enabling rapid engagement and disengagement in response to kinetochore attachment status. In contrast, the SPOC pathway exhibits more balanced sensitivity across multiple parameters, supporting a more gradual response to spindle alignment defects. These differences likely reflect the distinct temporal and spatial constraints under which these checkpoints operate during mitosis.

The potential for complex dynamics in strongly coupled regimes raises intriguing questions about the functional significance of non-linear behavior in cellular decision-making. While the existence of chaotic dynamics in biological checkpoint systems remains to be experimentally validated, the model provides testable predictions regarding the conditions under which such behavior might emerge. These include strong coupling between pathways, significant time delays in feedback loops, and intermediate values of spindle defect parameters.

From a methodological perspective, this work demonstrates the value of minimal models in understanding complex biological systems. By focusing on core interactions rather than comprehensive molecular details, I have derived insights into the essential design principles governing checkpoint function and crosstalk. This approach not only improves computational tractability but also helps identify the fundamental mechanisms that are conserved across species despite differences in molecular implementation.

The mathematical model of SAC-SPOC crosstalk generates several testable predictions that could guide future experimental studies. To validate the existence of the four operational regimes identified in the analysis, experiments could employ combinations of chemical inhibitors and genetic manipulations that target specific components of each pathway. For example, partial inhibition of Cdc5 kinase activity using analog-sensitive mutants (Cdc5-as) combined with varying doses of microtubule-targeting drugs like nocodazole could help delineate the boundaries between SAC-dominant and dual-checkpoint arrest states. Similarly, the SPOC-dominant regime could be explored using spindle misorientation techniques such as dynein motor protein mutations (dyn1$\Delta$) while maintaining proper kinetochore attachments. The model also predicts differential sensitivity profiles between SAC and SPOC pathways, which could be tested through quantitative proteomics approaches measuring checkpoint component abundance across various perturbation conditions. Live-cell imaging using fluorescently tagged Securin and Cyclin B reporters would enable direct observation of the predicted rheostat-like degradation dynamics and their response to different checkpoint states. These experimental approaches would not only validate the current model but potentially reveal additional complexities in checkpoint integration that could inform more sophisticated mathematical frameworks.

Several limitations of the current model should be acknowledged. First, spatial aspects of checkpoint signaling, such as the localization of components to different cellular compartments, are not explicitly represented. Second, the model primarily focuses on budding yeast, where both SAC and SPOC have been well-characterized, and extension to mammalian systems would require additional considerations. Third, while the in silico mutation analyses provide validation for key model predictions, further experimental testing is needed to fully assess the model’s accuracy.

While the model focuses on budding yeast, where both SAC and SPOC have been well-characterized, the underlying principles of checkpoint integration may have broader implications for metazoan cell division. In mammalian cells, the spindle assembly checkpoint is more complex, involving additional proteins like BubR1 and Mad3^14,51^. However, the core mechanism of tension-dependent checkpoint silencing remains conserved. Although a direct SPOC analog has not been identified in mammalian systems, similar spindle positioning mechanisms exist, particularly in asymmetrically dividing cells such as neural stem cells^5^. The minimal modeling approach suggests that even seemingly complex checkpoint behaviors can emerge from relatively simple reaction networks. Future work could extend this framework to explore checkpoint dynamics in more complex mammalian cell division contexts, potentially providing insights into mitotic errors associated with cancer and developmental disorders.

To address these limitations, several promising directions for model extension exist. Incorporating spatial modeling approaches would provide insights into how subcellular localization of checkpoint components influences pathway cross-regulation. Expanding the model to include post-translational modifications-particularly phosphorylation cascades mediated by Aurora B and Mps1 kinases-would enhance biological realism. Developing mammalian-specific versions that incorporate BubR1, p31$^{comet}$, and other metazoan-specific factors would facilitate translation of these findings to human cell division contexts. Rigorous experimental validation using tension manipulation techniques (such as controlled laser ablation of kinetochore-microtubule attachments) would test the model’s predictions regarding the four operational regimes. Finally, integrating this checkpoint model with existing cell cycle models would create a more comprehensive framework for understanding mitotic regulation in both normal and pathological contexts.

## Conclusion

The integrated mathematical analysis of SAC-SPOC crosstalk has revealed fundamental principles governing mitotic checkpoint coordination. By demonstrating that deterministic models with linear tension-sensing mechanisms generate ultrasensitivity and bistability without stochasticity, I challenge previous assumptions about checkpoint regulation.

A central discovery of this work is the coexistence of two distinct regulatory modes within the system: at the molecular level, core components like the Mad2-Cdc20-APC/C interaction network function as a bistable toggle switch with clearly defined activation thresholds, while the downstream degradation of substrates like Securin and Cyclin B exhibits rheostat-like, progressive dynamics. This reconciles the seemingly paradoxical observations of Dick et al^48^. and Cirillo et al^49^., who noted both switch-like and gradual behaviors within the same system.

The computational mutation analysis provides strong support for the model’s biological relevance. Perturbations of Mad2 concentration predictably altered APC/C activation kinetics, while modulation of Bfa1/Bub2 affected Tem1 regulation in patterns consistent with experimental data. Particularly revealing was the response to varying Cdc5 levels, which simultaneously influenced both checkpoint pathways, confirming its role as a central coordinator of checkpoint signaling. These in silico experiments reproduce key phenotypes observed in laboratory studies without parameter adjustments, validating the model’s predictive capacity.

The framework offers testable predictions, particularly regarding the four operational regimes I identified (checkpoint silence, SAC-dominant, SPOC-dominant, and dual-checkpoint arrest). The mathematical framework generates several testable predictions that warrant experimental validation. The four operational regimes could be tested through specific experimental interventions: wild-type cells (checkpoint silence), low-dose nocodazole treatment (SAC-dominant arrest), *dyn1*$\Delta$/*kar9*$\Delta$ mutations (SPOC-dominant arrest), and combined nocodazole with *dyn1*$\Delta$ (dual-checkpoint arrest). The predicted enhanced robustness of dual-checkpoint arrest could be assessed by measuring mitotic timing variability across these conditions. Single-cell fluorescence microscopy with Securin-GFP and Mad2-mCherry would enable simultaneous observation of toggle switch dynamics and rheostat behavior. Finally, analog-sensitive Cdc5 mutants (Cdc5-as) could validate the role of Cdc5 as a critical mediator of checkpoint crosstalk. These experiments would provide critical tests of the model’s key predictions and potentially reveal new insights into checkpoint coordination during mitosis.

Beyond theoretical insights, the model has implications for understanding checkpoint dysregulation in diseases characterized by chromosomal instability. The differential sensitivity profiles of SAC and SPOC pathways suggest potential vulnerabilities that could be exploited therapeutically, particularly in cancer contexts where checkpoint adaptation mechanisms are often altered.

Future extensions should incorporate spatial aspects of checkpoint signaling and expand the framework to mammalian systems, where additional regulatory components add complexity to the core mechanisms described here. By establishing the minimal requirements for robust checkpoint integration, this work provides a foundation for understanding how cells ensure faithful chromosome segregation through coordinated surveillance systems.

In conclusion, the mathematical model of SAC-SPOC crosstalk advances the understanding of how mitotic checkpoints cooperate to maintain genomic stability. By elucidating the principles governing this cooperation, this work provides insights into fundamental aspects of cell division control and offers a framework for investigating checkpoint dysregulation in disease states such as cancer.

## Materials and methods

### Mathematical framework and model assumptions

I constructed a deterministic model based on ordinary differential equations (ODEs) to represent the interaction between the Spindle Assembly Checkpoint (SAC) and the Spindle Position Checkpoint (SPOC) in budding yeast. The model integrates key components from each pathway and focuses on their essential regulatory interactions.

Rather than modeling every molecular detail, I adopted a reductionist approach that captures the functional essence of these checkpoint systems. This simplification offers two key advantages: mathematical tractability and clearer insight into fundamental regulatory principles.

The model assumes:

- Mass-action kinetics for all reactions
- Conservation of total protein amounts
- Effective rate constants representing compartmentalized interactions
- Rapid equilibrium approximations for complex formation
- Linear tension dependence on kinetochore attachment

Key derived parameters used in the analysis:

- $\gamma$ (coupling strength): Defined as $\gamma = [Cdc5]/[Cdc5]_0$, where $[Cdc5]_0$ is the basal Cdc5 concentration. This represents the relative strength of crosstalk between SAC and SPOC pathways.
- $\tau$ (time delay): Defined as $\tau = 1/(k_{cat} + k_{di})$, representing the characteristic time scale for checkpoint complex dissociation and signal propagation.

The model inputs are the attachment status of kinetochores (KinA) and spindle pole alignment (SpinA), which serve as the primary sensors for the SAC and SPOC systems, respectively.

### Parameter selection and validation

Model parameters were derived from a combination of experimental measurements and optimization approaches^52^. Initial parameter values were selected based on published kinetic studies of checkpoint components in budding yeast, particularly from quantitative proteomic and live-cell imaging data from Musacchio and Salmon (2007) and Caydasi et al. (2012). Where direct measurements were unavailable, I employed constraints-based parameter estimation to ensure physiologically plausible values.

The parameter set was validated by comparing model predictions against experimental observations of checkpoint dynamics in both wild-type and mutant conditions. In particular, I focused on matching the experimentally observed timescales of anaphase onset (approximately 20-30 minutes after checkpoint activation) and the characteristic degradation profiles of key substrates (Securin and Cyclin B). The model reproduced experimentally observed phenotypes of specific checkpoint mutations (e.g., Mad2 depletion, Bfa1 overexpression) without requiring adjustment of other parameters, providing strong support for its biological relevance.

Sensitivity analysis (as shown in Figure 5) confirmed that the conclusions are robust to moderate parameter variations, with the system maintaining its essential dynamical features across a substantial region of parameter space. All simulations were performed in physiologically relevant concentration ranges based on quantitative proteomics data.

### Dynamical model equations

The SAC-SPOC system is described by a set of coupled ODEs representing the time evolution of key system components:

21 $$\begin{aligned} \frac{d[MCCT]}{dt}&= k_a([KinA_{total}]-[KinA])([Mad2T] - [MCCT]) \nonumber \\&- k_{cat} \cdot [KinA] \cdot ([APCT] - [APCMCC]) \cdot [APCMCC] \nonumber \\&- [Cdc5] \cdot [APCMCC] \end{aligned}$$

Where MCCT represents the Mitotic Checkpoint Complex, which inhibits the Anaphase-Promoting Complex (APC/C). The first term describes MCC formation catalyzed by unattached kinetochores, while the second and third terms capture MCC dissolution through APC/C-mediated and Cdc5-mediated pathways, respectively.

For the SPOC pathway, similar dynamics govern the inhibitory Bfa1Bub2 complex:

22 $$\begin{aligned} \frac{d[Bfa1Bub2T]}{dt}&= k_a([SpinA_{total}] - [SpinA])([Bub2T] - [Bfa1Bub2T]) \nonumber \\&- k_{cat} \cdot [SpinA] \cdot ([Tem1T] - [Bfa1Bub2Tem1]) \cdot [Bfa1Bub2Tem1] \nonumber \\&- [Cdc5] \cdot [Bfa1Bub2Tem1] \end{aligned}$$

The kinetochore attachment and spindle alignment dynamics are modeled with simple first-order kinetics:

23 $$\begin{aligned} \frac{d[KinA]}{dt}&= 0.1 \cdot ([KinA_{total}]/2 - [KinA]) + 0.05 \cdot [MCCT] \end{aligned}$$

24 $$\begin{aligned} \frac{d[SpinA]}{dt}&= 0.1 \cdot ([SpinA_{total}]/2 - [SpinA]) + 0.05 \cdot [Bfa1Bub2T] \end{aligned}$$

Equilibrium concentrations for protein complexes are calculated using quadratic formulas derived from mass-action principles:

25 $$\begin{aligned} [APCMCC]&= \frac{K_m + [APCT] + [MCCT] - \sqrt{(K_m + [APCT] + [MCCT])^2 - 4[MCCT][APCT]}}{2} \end{aligned}$$

26 $$\begin{aligned} [Bfa1Bub2Tem1]&= \frac{K_m + [Tem1T] + [Bfa1Bub2T] - \sqrt{(K_m + [Tem1T] + [Bfa1Bub2T])^2 - 4[Bfa1Bub2T][Tem1T]}}{2} \end{aligned}$$

where $K_m = (k_{di} + k_{cat})/k_{as}$ represents the effective dissociation constant.

### Linear tension model analysis

The linear tension model inherently generates ultrasensitivity in the Spindle Assembly Checkpoint system without requiring stochasticity. The key differential equation governing APC dynamics:

27 $$\begin{aligned} \frac{d[APC]}{dt} = -k_{inhib} \cdot [APC] \cdot [MCC] + k_{decay} \cdot [APC\text {-}MCC] + k_{silencing} \cdot [APC\text {-}MCC] \cdot [tension] \end{aligned}$$

With the linear tension model defined as:

28 $$\begin{aligned} [tension] = [KinA] \end{aligned}$$

For the APC-MCC complex, I have:

29 $$\begin{aligned} \frac{d[APC\text {-}MCC]}{dt} = k_{inhib} \cdot [APC] \cdot [MCC] - k_{decay} \cdot [APC\text {-}MCC] - k_{silencing} \cdot [APC\text {-}MCC] \cdot [KinA] \end{aligned}$$

At steady state, $\frac{d[APC]}{dt} = \frac{d[APC\text {-}MCC]}{dt} = 0$, which gives:

30 $$\begin{aligned} k_{inhib} \cdot [APC] \cdot [MCC] = k_{decay} \cdot [APC\text {-}MCC] + k_{silencing} \cdot [APC\text {-}MCC] \cdot [KinA] \end{aligned}$$

With mass conservation:

31 $$\begin{aligned} [APC]_{total} = [APC] + [APC\text {-}MCC] \end{aligned}$$

Solving for steady-state free APC concentration:

32 $$\begin{aligned} [APC]_{ss} = [APC]_{total} \cdot \frac{k_{silencing} \cdot [KinA] + k_{decay}}{k_{silencing} \cdot [KinA] + k_{decay} + k_{inhib} \cdot [MCC]} \end{aligned}$$

When $k_{inhib} \cdot [MCC] \gg k_{decay}$, this simplifies to:

33 $$\begin{aligned} [APC]_{ss} \approx [APC]_{total} \cdot \frac{k_{silencing} \cdot [KinA]}{k_{silencing} \cdot [KinA] + k_{inhib} \cdot [MCC]} \end{aligned}$$

This has the mathematical form of a Hill function with coefficient $n> 1$:

34 $$\begin{aligned} [APC]_{ss} = [APC]_{total} \cdot \frac{K^n}{K^n + \theta ^n} \end{aligned}$$

Where $$K = [KinA]$$ and $$\theta = \frac{k_{inhib} \cdot [MCC]}{k_{silencing}}$$.

For small [*KinA*]: $[APC]_{ss} \approx 0$ For large [*KinA*]: $[APC]_{ss} \approx [APC]_{total}$

The bifurcation analysis confirms this with a measured Hill coefficient of approximately 4.3, demonstrating that the deterministic linear tension model inherently generates ultrasensitivity without requiring stochastic terms in the KinU$\rightarrow$KinA transition.

### Numerical simulation and analysis

The ODE system was solved numerically using MATLAB R2024a (MathWorks, Natick, MA) with the ode45 solver for non-stiff problems and ode15s for stiff cases. High accuracy tolerances were employed (RelTol = $10^{-8}$, AbsTol = $10^{-10}$) to ensure reliable capture of threshold behaviors. Bifurcation diagrams were generated by scanning over a range of key parameters and solving for steady states using a root-finding algorithm (fzero). Stability was assessed by examining the sign of the local derivative at each steady state, with eigenvalue analysis for multi-dimensional stability assessment.

For sensitivity analyses, I systematically varied individual parameters while keeping all others constant (Table 3), and examined the resulting changes in system behavior, particularly focusing on the position and width of bistable regions. Global sensitivity indices were calculated using a variance-based approach across the parameter space. For stochastic simulations, we implemented a continuous-time approach with noise terms proportional to the square root of concentration values, which is more appropriate for biochemical systems than discrete Gillespie methods. This approach models the intrinsic noise of the reaction system while maintaining the continuous nature of protein concentration changes. The stochastic differential equations take the form:

35 $$\begin{aligned} dX_i = f_i(X) dt + \sigma \sqrt{X_i} dW_i \end{aligned}$$

where $f_i(X)$ represents the deterministic dynamics from our ODE system, $\sigma$ is the noise strength parameter (varied from 0.001 to 0.1), $X_i$ are the concentration variables, and $dW_i$ are independent Wiener processes. Simulations employed the Euler-Maruyama method with adaptive time steps.

For chaos detection, I employed multiple complementary methods including Lyapunov exponent calculation, correlation dimension analysis, and Poincaré sections. Parameter space exploration utilized Latin Hypercube sampling for efficient coverage of high-dimensional parameter spaces, with over 1,500 parameter combinations tested.

### Experimental validation

To validate the minimal model, I compared simulation predictions against published experimental datasets. For the SAC dynamics, I used quantitative data from Dick et al.^48^ on Securin degradation kinetics in both wild-type and Mad2-depleted cells. The model predictions showed excellent agreement with these measurements, achieving correlation coefficients (R²) of 0.91 for wild-type conditions and 0.88 for Mad2 siRNA knockdown scenarios.

Additionally, I validated the integrated checkpoint model against recent spatiotemporal data from Cirillo et al.^49^ on Cyclin B degradation patterns. The model accurately reproduced the characteristic degradation profiles observed under various experimental conditions, including wild-type, Mad2 siRNA knockdown, and Aurora B inhibitor treatments. This dual validation using two independent APC/C substrates (Securin and Cyclin B) provides strong support for the model’s ability to capture essential features of checkpoint signaling and silencing.

Parameter sensitivity analysis confirmed that the conclusions remain robust to moderate variations (±20%) in key rate constants, with the system maintaining its characteristic ultrasensitivity and four operational regimes across physiologically relevant parameter ranges.

### Computational resources and numerical implementation

All simulations and analyses were performed using MATLAB R2024a (MathWorks, Natick, MA) on a workstation with an Intel Core i7-11800H processor (2.30 GHz, 16 cores) and 64 GB RAM running Windows 11 Pro. The deterministic ODE system was solved using the ode45 solver for non-stiff problems and ode15s for stiff cases, with relative tolerance set to $10^{-8}$ and absolute tolerance to $10^{-10}$ to ensure high numerical accuracy.

For stochastic simulations, I implemented a continuous-time approach with noise terms proportional to the square root of concentration values, which is more appropriate for biochemical systems than discrete Gillespie methods. This approach models the intrinsic noise of the reaction system while maintaining the continuous nature of protein concentration changes. Simulations employed the Euler-Maruyama method with adaptive time steps.

For chaos detection, I employed multiple complementary methods including Lyapunov exponent calculation, correlation dimension analysis, and Poincaré sections. The largest Lyapunov exponent was calculated using a direct method that tracks the divergence of nearby trajectories, while correlation dimension was estimated using the Grassberger-Procaccia algorithm. These analyses were conducted across an extensive parameter space, with over 1,500 parameter combinations explored using Latin Hypercube sampling for efficient coverage of the high-dimensional parameter space.

Bifurcation analyses were performed using custom-built continuation algorithms, with solution branches tracked through limit points. Data visualization was performed using MATLAB’s plotting functions with custom color schemes optimized for clarity and accessibility. All code and parameter sets are available upon request from the corresponding author.

### Model parameters

See Table 3.

**Table 3:** Complete Model Parameters, Initial Conditions, and Their Biological Interpretation.

Parameter	Value	Initial Conc.	Units	Description
$k_{1f}$	4.0	[Mad2] = 1.0	$\text {molecule}^{-1}\text {s}^{-1}$	Mad2-Cdc20 binding rate
$k_{1b}$	0.05	[Cdc20] = 1.2	$\text {s}^{-1}$	Mad2-Cdc20 dissociation rate
$k_{2f}$	0.9	[Mad2Cdc20] = 0.0	$\text {molecule}^{-1}\text {s}^{-1}$	MCC formation rate
$k_{2b}$	0.09	[Bub3BubR1] = 1.0	$\text {s}^{-1}$	MCC dissociation rate
$k_{3f}$	0.01	[MCC] = 0.0	$\text {molecule}^{-1}\text {s}^{-1}$	MCC-APC/C binding rate
$k_{3b}$	0.001	[APC/C] = 1.2	$\text {s}^{-1}$	MCC-APC/C dissociation rate
$k_{4f}$	10.0	[MCCAPC/C] = 0.0	$\text {molecule}^{-1}\text {s}^{-1}$	APC/C-Cdc20 binding rate
$k_{4b}$	0.0001	[APC/C-Cdc20] = 0.0	$\text {s}^{-1}$	APC/C-Cdc20 dissociation rate
$k_{6f}$	3.5	[Bfa1Bub2] = 0.6	$\text {s}^{-1}$	Bfa1Bub2 activation rate
$k_{6b}$	0.01	[Bfa1Bub2*] = 0.0	$\text {s}^{-1}$	Bfa1Bub2 inactivation rate
$k_{7f}$	0.91	[Tem1] = 0.1	$\text {molecule}^{-1}\text {s}^{-1}$	Tem1 binding rate
$k_{7b}$	0.01	[Bfa1Bub2Tem1] = 0.0	$\text {s}^{-1}$	Tem1 dissociation rate
$k_{8f}$	2.0		$\text {molecule}^{-1}\text {s}^{-1}$	Crosstalk binding rate
$k_{8b}$	0.05		$\text {s}^{-1}$	Crosstalk dissociation rate
$k_{\text {attach}}$	0.25	[KinU] = 92.0	$\text {s}^{-1}$	Kinetochore attachment rate
$k_{\text {align}}$	0.4	[KinA] = 0.0	$\text {s}^{-1}$	Spindle alignment rate
$k_{\text {detach}}$	0.001	[SpbM] = 2.0	$\text {s}^{-1}$	Kinetochore detachment rate
$k_{\text {misalign}}$	0.001	[SpbA] = 0.0	$\text {s}^{-1}$	Spindle misalignment rate
$k_{\text {diss1}}$	0.2		$\text {s}^{-1}$	KinA-mediated Mad2Cdc20 dissociation
$k_{\text {diss2}}$	0.1		$\text {s}^{-1}$	KinA-mediated MCC dissociation
$k_{\text {diss3}}$	0.05		$\text {s}^{-1}$	KinA-mediated MCCAPC/C dissociation
$k_{\text {diss4}}$	0.01		$\text {s}^{-1}$	SpbA-mediated complex dissociation
$k_{\text {diss5}}$	0.01		$\text {s}^{-1}$	Checkpoint-mediated crosstalk dissociation
		[Cdc5] = 0.1	molecule	Polo kinase concentration
		[Kin4] = 0.05	molecule	Regulatory kinase concentration
Threshold	0.85			KinA ratio activation threshold
Hill coefficient	8			Activation response steepness

## Data Availability

All data generated or analyzed during this study are included in the published article and its supplementary information files. Raw data and additional resources are available from the corresponding author upon request.
